# Molecular repertoire of *Deinococcus radiodurans* after 1 year of exposure outside the International Space Station within the Tanpopo mission

**DOI:** 10.1186/s40168-020-00927-5

**Published:** 2020-10-29

**Authors:** Emanuel Ott, Yuko Kawaguchi, Denise Kölbl, Elke Rabbow, Petra Rettberg, Maximilian Mora, Christine Moissl-Eichinger, Wolfram Weckwerth, Akihiko Yamagishi, Tetyana Milojevic

**Affiliations:** 1 Space Biochemistry Group, Department of Biophysical Chemistry, University of Vienna, Vienna, Austria; 2 Planetary Exploration Research Center (PERC), Chiba Institute of Technology (CIT), Chiba, Japan; 3 Institute of Aerospace Medicine, Radiation Biology Department, German Aerospace Center, Cologne, Germany; 4 Department of Internal Medicine, Section of Infectious Diseases and Tropical Medicine, Medical University Graz, Graz, Austria; 5 Department of Ecogenomics and Systems Biology, University of Vienna, Vienna, Austria; 6 Vienna Metabolomics Center (VIME), University of Vienna, Vienna, Austria; 7 Department of Life Science, Tokyo Institute of Technology, Nagatsuta, Yokohama, Japan

**Keywords:** *Deinococcus radiodurans*, Low earth orbit, Proteomics, Transcriptomics, Metabolomics, Molecular stress response

## Abstract

**Background:**

The extraordinarily resistant bacterium *Deinococcus radiodurans* withstands harsh environmental conditions present in outer space. *Deinococcus radiodurans* was exposed for 1 year outside the International Space Station within Tanpopo orbital mission to investigate microbial survival and space travel. In addition, a ground-based simulation experiment with conditions, mirroring those from low Earth orbit, was performed.

**Methods:**

We monitored *Deinococcus radiodurans* cells during early stage of recovery after low Earth orbit exposure using electron microscopy tools. Furthermore, proteomic, transcriptomic and metabolomic analyses were performed to identify molecular mechanisms responsible for the survival of *Deinococcus radiodurans* in low Earth orbit.

**Results:**

*D. radiodurans* cells exposed to low Earth orbit conditions do not exhibit any morphological damage. However, an accumulation of numerous outer-membrane-associated vesicles was observed. On levels of proteins and transcripts, a multi-faceted response was detected to alleviate cell stress. The UvrABC endonuclease excision repair mechanism was triggered to cope with DNA damage. Defense against reactive oxygen species is mirrored by the increased abundance of catalases and is accompanied by the increased abundance of putrescine, which works as reactive oxygen species scavenging molecule. In addition, several proteins and mRNAs, responsible for regulatory and transporting functions showed increased abundances. The decrease in primary metabolites indicates alternations in the energy status, which is needed to repair damaged molecules.

**Conclusion:**

Low Earth orbit induced molecular rearrangements trigger multiple components of metabolic stress response and regulatory networks in exposed microbial cells. Presented results show that the non-sporulating bacterium *Deinococcus radiodurans* survived long-term low Earth orbit exposure if wavelength below 200 nm are not present, which mirrors the UV spectrum of Mars, where CO_2_ effectively provides a shield below 190 nm. These results should be considered in the context of planetary protection concerns and the development of new sterilization techniques for future space missions.

Video Abstract

## Introduction

As humans continue to conquer the realms of the solar system, understanding the molecular mechanisms of survival in outer space becomes increasingly important. Outer space is a hostile environment, which constrains any form of life. Remarkably, a few extremophilic microbial species have been shown to withstand the drastic influence of the outer space factors [1–5]. Exposed to the outer space environment, microorganisms are challenged by several hostile parameters: galactic cosmic and solar UV radiation, extreme vacuum, temperature fluctuations, desiccation, freezing, and microgravity. The International Space Station (ISS) provides a suitable environment for astrobiological experiments in the low Earth orbit (LEO). Limited previous studies partially described microbial responses after exposure outside the ISS [6–10]. Significantly impacting the development of astrobiology, the EXPOSE experiments (2008–2015) concluded that not only spore-forming bacteria such as *Bacillus subtilis* can survive an interplanetary travel, but also seeds, lichens (e.g., *Stichococcus sp*, *Trichoderma sp* and *Acarospora sp*) [10–12], and non-spore forming thermophilic bacteria such as *Deinococcus geothermalis* [13]. However, we have still been missing an explicit knowledge of molecular mechanisms permitting survival and adaptation in the outer space environment. Space parameters affect microorganisms by altering a variety of physiological features, including proliferation rate, cell metabolism, cell division, cell motility, virulence, drug resistance, and biofilm production [9, 10, 14–16]. These aforementioned physiological perturbations of space-exposed microorganisms are very poorly understood on the molecular level. In order to achieve a detailed understanding of the full-functional molecular set up of microorganisms exposed to outer space, a comprehensive multi-omics analysis of their molecular responses is desirable.

The gram-positive bacterium *Deinococcus radiodurans* possesses numerous remarkable properties [17–19], which made it a suitable candidate for long-term space exposure experiments within the scope of the Tanpopo orbital project [20–22]. Preliminary experiments with monochromatic lamps at UV_172 nm_ UV_254 nm,_ indicated that cells of *D. radiodurans* should be able to withstand solar UV radiation on the ISS for 1 year as multi-layers of more than 200 μm thickness [21]. Additionally, several initial investigations prior to the Tanpopo space mission already resolved molecular responses of *D. radiodurans* to selected simulated parameters of the outer space environment [23, 24]. Exposure to extreme conditions such as ionizing radiation, UV radiation and desiccation cause an increase of reactive oxygen species, which severely damage nucleic acids, by forming bipyrimidine dimers or introducing DNA double strand breaks. It is noteworthy that the number of nucleic acid fragmentations of *D. radiodurans* and *E. coli* when exposed to the same amount of radiation do not differentiate. However, with a dose of ionizing radiation that is already lethal to *E. coli*, when exposed to it for 3-4 hours, an efficient protection and repair mechanism in *D. radiodurans* allows the nucleic acid fragments in *D. radiodurans* to be reassembled into complete chromosomes and the cells return to normal growth [25]. Although the nucleic acid repair mechanisms show no remarkable functional difference in *E. coli* [26], *D. radiodurans* is 50 times more resistant to ionizing radiation and 33 times more resistant to UVC radiation [27]. The mechanisms which allow *D. radiodurans* to survive under such conditions are not completely unraveled yet. However, research data suggests that the extent of protein damage caused by irradiation is related to the cells survival potential, which suggests that *D. radiodurans* most likely focuses on protecting proteins from oxidative damage [28]. Possible explanations for an increased protein protection are an increased ROS-scavenging and ROS-detoxifying activity via orthophosphate-manganese-small molecule complexes [29], and a higher intracellular manganese to iron ratio than in radiation sensitive-microorganisms [30, 31]. Though, these physiological and metabolic adaptions are unique for *D. radiodurans*, it is hard to explain genome reconstitution without considering DNA repair pathways [25]. Generally, in prokaryotes, there are several DNA repair systems, such as photoreactivation, nucleotide excision repair, base excision repair, mismatch repair, double strand break repair, homology directed repair and ultraviolet damage endonuclease repair, each specialized to a certain type of damage. Nucleotide excision repair stands out from the other repair mechanisms, since it is able to recognize a broad range of structurally unrelated DNA damages [32]. It consists of four crucial proteins, UvrA, UvrB, UvrC and UvrD, which in conjunction with each other orchestrate effective DNA repair performance. The Uvr cluster proteins are triggered by the exposure of *D. radiodurans* to several space-related conditions (UVC and vacuum), albeit the response to these factors is a multi-layered process, in which many other molecular components, in addition to DNA damage repair, are involved [23, 24].

In this study, dehydrated cells of *D. radiodurans* were exposed to LEO conditions for 1 year outside the ISS in frames of the Tanpopo space mission [33]. After exposure and subsequent recovery in complex medium, metabolites, proteins and mRNAs were extracted from space-exposed cells, analyzed with integrative –omics techniques and compared to ground controls. The results show the early molecular response of *D. radiodurans* after LEO exposure, help to understand which molecular tools are used to cope with the damage induced by outer space conditions and highlight the power of combining different –omics techniques to unravel molecular stress response mechanisms.

## Results

### Survival and post-exposure analysis

**Fig. 1:**
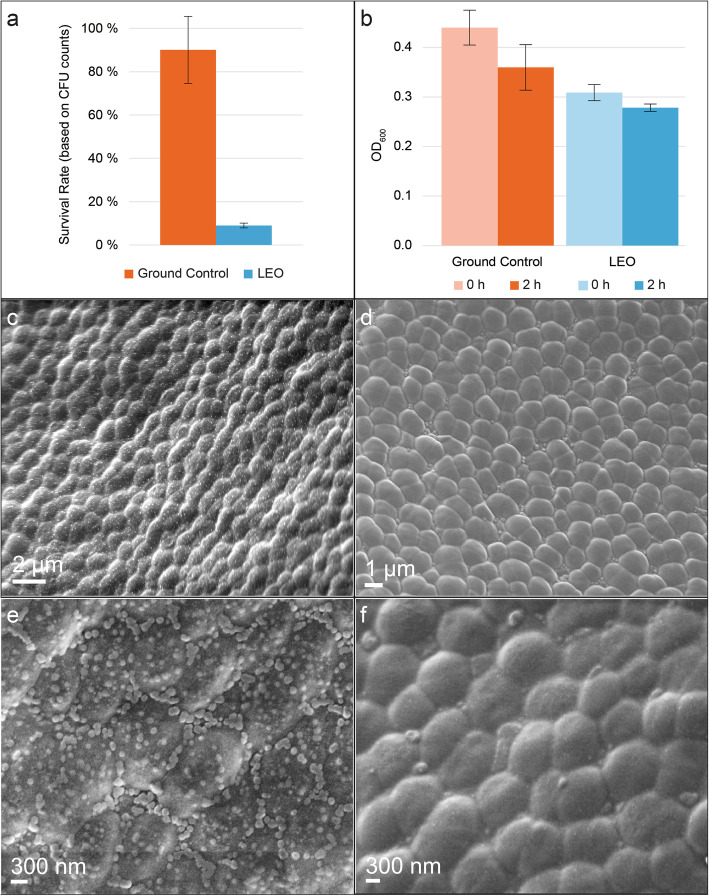
Survival and post-exposure analysis. **a** Survival rate of LEO exposed and ground control cells based on colony forming units counting. **b** OD_600_ measurements of exposed and control cells at *t*_0_ and after 2 h of recovery in complex medium. **c**–**f** Scanning electron microscopy (SEM) images showing the upper surface of multi-layers of dehydrated cells of *D. radiodurans* deposited on aluminum plates. **c**, **e** SEM images of *D. radiodurans* cells exposed to LEO in Tanpopo mission. **d**, **f** SEM images of ground control cells of *D. radiodurans*. **e**, **f** Higher magnification SEM images displaying upper surface of multi-layers of dehydrated cells of *D. radiodurans*. Error bars show the error-represented standard deviation, respectively, of *n* = 3 biological replicates

Dehydrated *D. radiodurans* cells survived exposure to the low Earth orbit for 1 year (Fig. 1a); however, decreased survival rates compared to ground controls kept on Earth under dehydrated conditions during the exposure time (Fig. 1a), were observed. Survival rates were calculated by colony forming units as N/N_0_, where N is the number of CFU’s/mL after 1 year and *N*_0_ is the number of CFU’s/mL of the stock before dehydration. As survival of space-returned cells was confirmed successfully, a recovery time of 2 h was chosen to see the early response after exposure to harsh LEO conditions. The recovery time was chosen based on none-exposed, dried cells that already showed growth between three and six hours of recovery [24].

After the cells were allowed to recover for 2 h in complex medium, an insignificant decrease in OD_600_ (Fig. 1b) was observed, for both LEO exposed as well as ground control cells. In order to investigate cellular integrity after a long-term LEO exposure, the surface of dehydrated, clustered cell layers of *D*. *radiodurans* deposited on aluminum plates, was examined via scanning electron microscopy (SEM). The surface of dehydrated *D*. *radiodurans* cells showed no detectable damage and preserved its integrity after LEO exposure. However, SEM observations revealed the accumulation of multiple nano-sized particles over the surface of LEO-returned cells (Fig. 1c–f; Fig. S1 and S2). These spherical-like morphologies were not visible on the surface of ground control cells and could be attributed to the results of direct influence of LEO parameters on biological material, e.g., Maillard reactions [24, 34].

**Fig 2.**
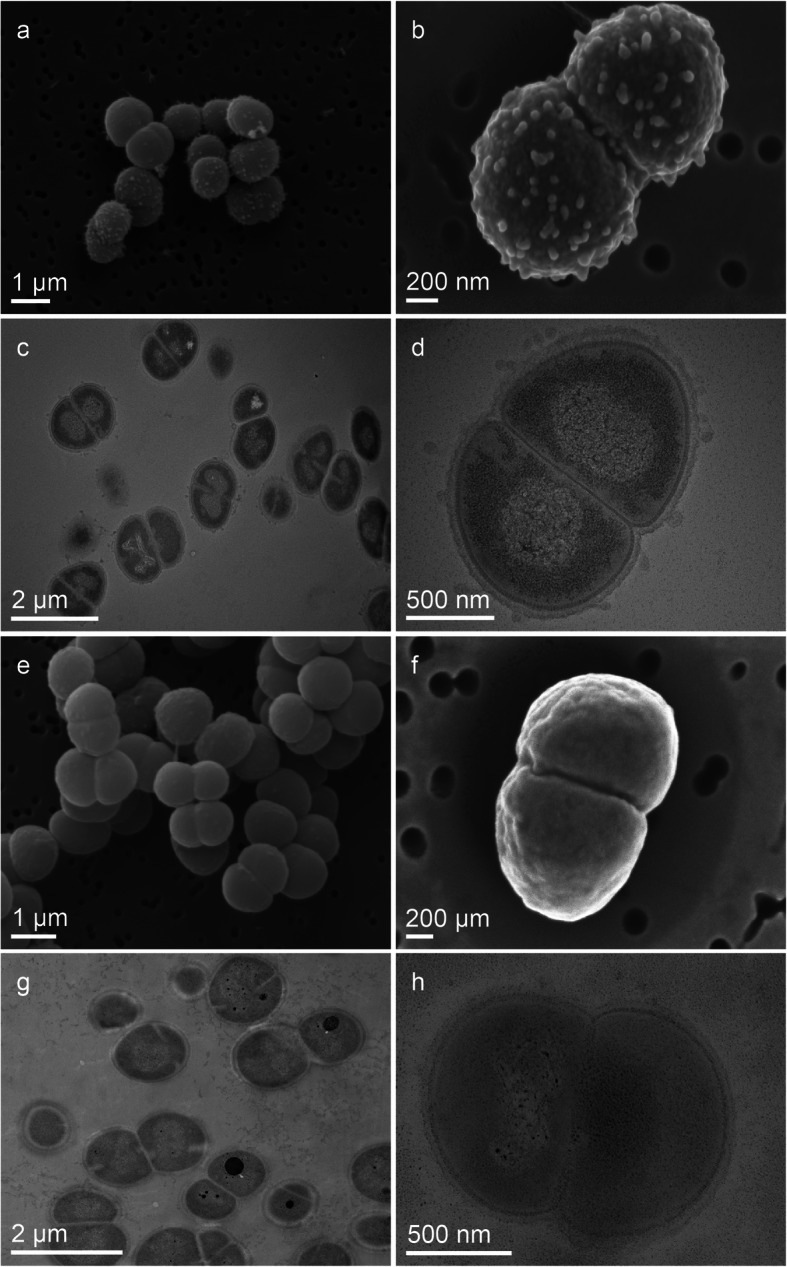
Scanning and transmission electron microscopy (SEM and TEM) images of *D. radiodurans* cells recovered after LEO exposure in complex medium. **a**, **b** SEM images of recovered *D. radiodurans* cells after LEO exposure. **c**, **d** TEM images of recovered *D. radiodurans* cells after LEO exposure. **e**, **f** SEM images of ground control *D. radiodurans* cells. **g**, **h** TEM images of ground control *D. radiodurans* cells

Subsequently, the morphology of space-returned, dehydrated, and non-exposed cells of *D. radiodurans* was inspected upon recovery in a liquid complex medium. The typical *D*. *radiodurans* morphology of diplococci and tetracocci is shown in Fig. 2 and Fig. S3. Compared to the ground control, cells in early stages of recovery after space exposure were characterized by cell surface-associated vesicular structures (Fig S3A). Investigations with transmission electron microscopy (TEM) confirmed this observation. Qualitative TEM observations of space-returned *D. radiodurans* cells revealed pronounced outer membrane-associated events with numerous vesicles accumulated around the cell surface (Fig. 2d). To obtain a comprehensive perspective of molecular changes induced by LEO exposure, we further examined the transcriptome, exoproteome, intracellular proteome, and metabolome responses of space-returned *D. radiodurans*.

### Low Earth orbit driven proteomic alterations

**Fig. 3:**
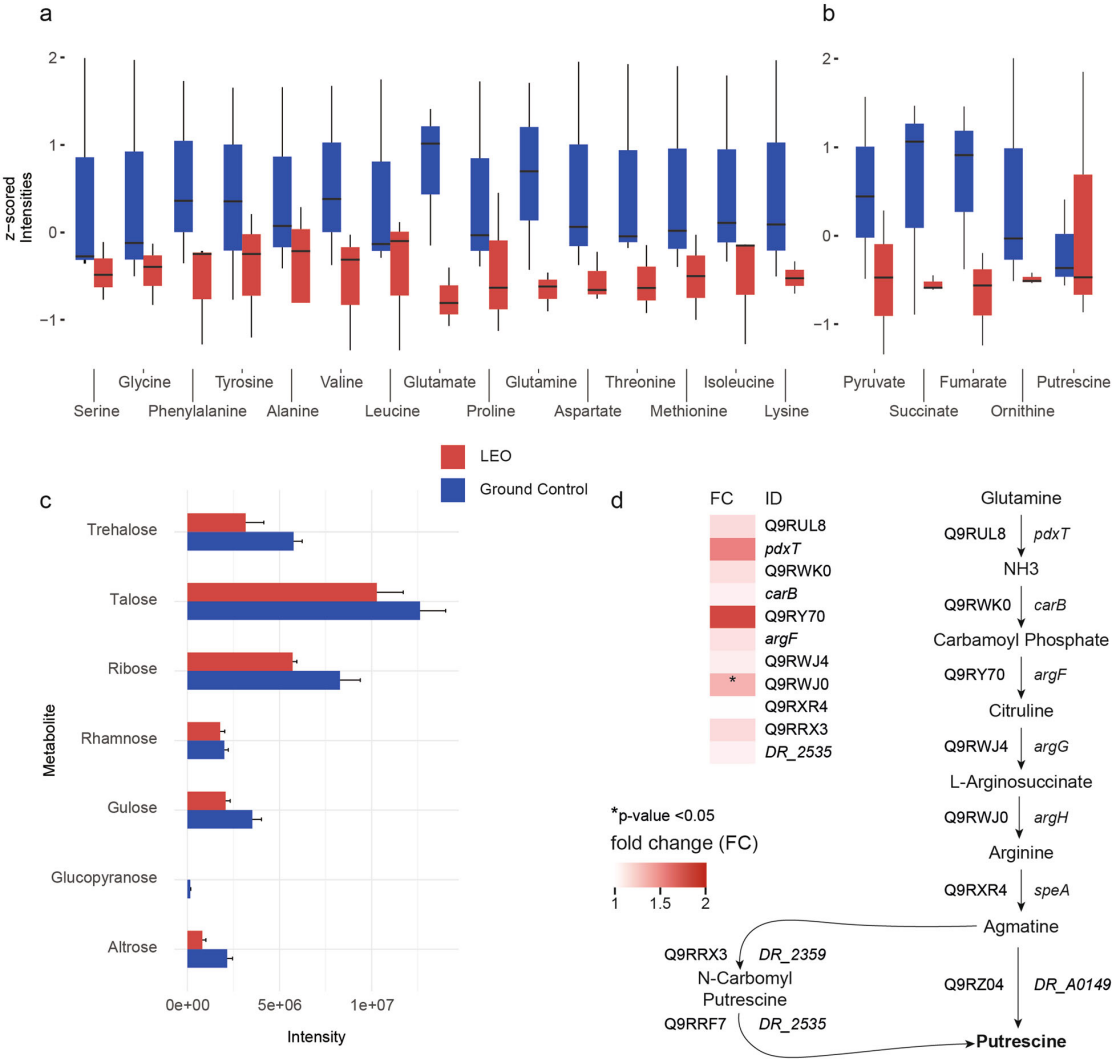
Metabolomic response after LEO exposure. **a**, **b** Results of the targeted metabolomics approach, illustrating amino acids (**a**) and organic acids and polyamine (precursors) (**b**). **c** Abundances of sugars identified via untargeted metabolomics approach. **d** Proteomics and transcriptomics data for the biosynthesis pathway from glutamine to putrescine. Proteins are indicated as Uniprot IDs and mRNAs are shown as *gene names* or *Locus tags*. Quantitative comparisons are based on fold changes (LEO/Ctrl), *p* values for proteomics and corrected *p* values (*q* values) for transcriptomics

**Fig. 4:**
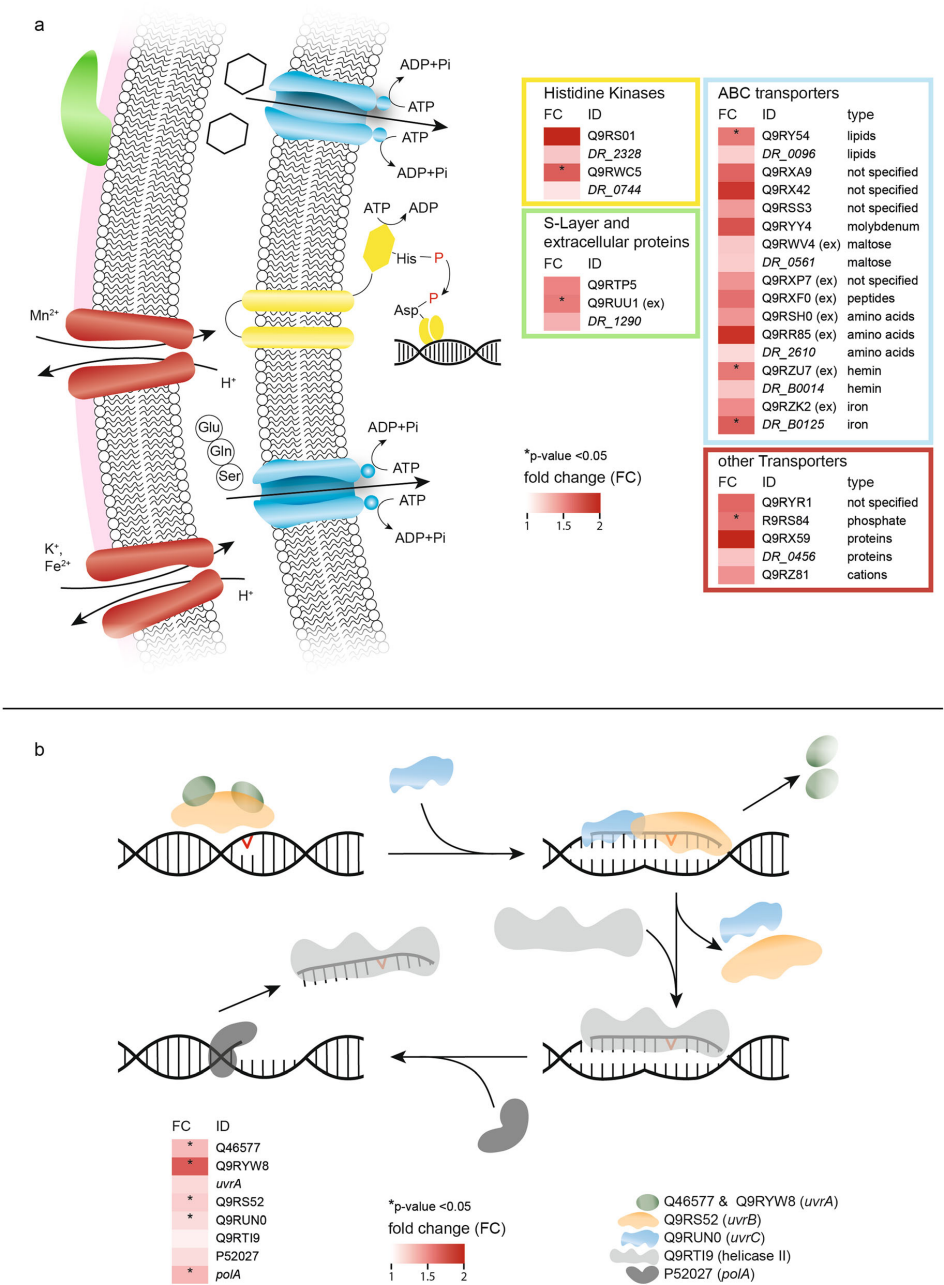
Proteotranscriptomic rearrangements as response to the LEO environment. **a** Membrane associated proteins/transporters/extracellular proteins which were identified higher abundant after LEO exposure in *D. radiodurans*. Color coding: yellow–histidine kinases; green–S-layer and extracellular proteins; blue–ABC transporters; red–other transporters. Proteins identified through intracellular and extracellular (ex) proteomics measurements are indicated as Uniprot IDs, transcriptomics data is shown as *Locus tags*. **b** Quantitative UvrABC nucleotide excision repair mechanism data in *D. radiodurans* after exposure to LEO. Intracellular proteomics data is shown as Uniprot IDs, transcriptomics data as *gene names*. Quantitative comparisons are based on fold changes (LEO/Ctrl), *p* values for proteomics and corrected *p* values (*q* values) for transcriptomics

**Fig 5.**
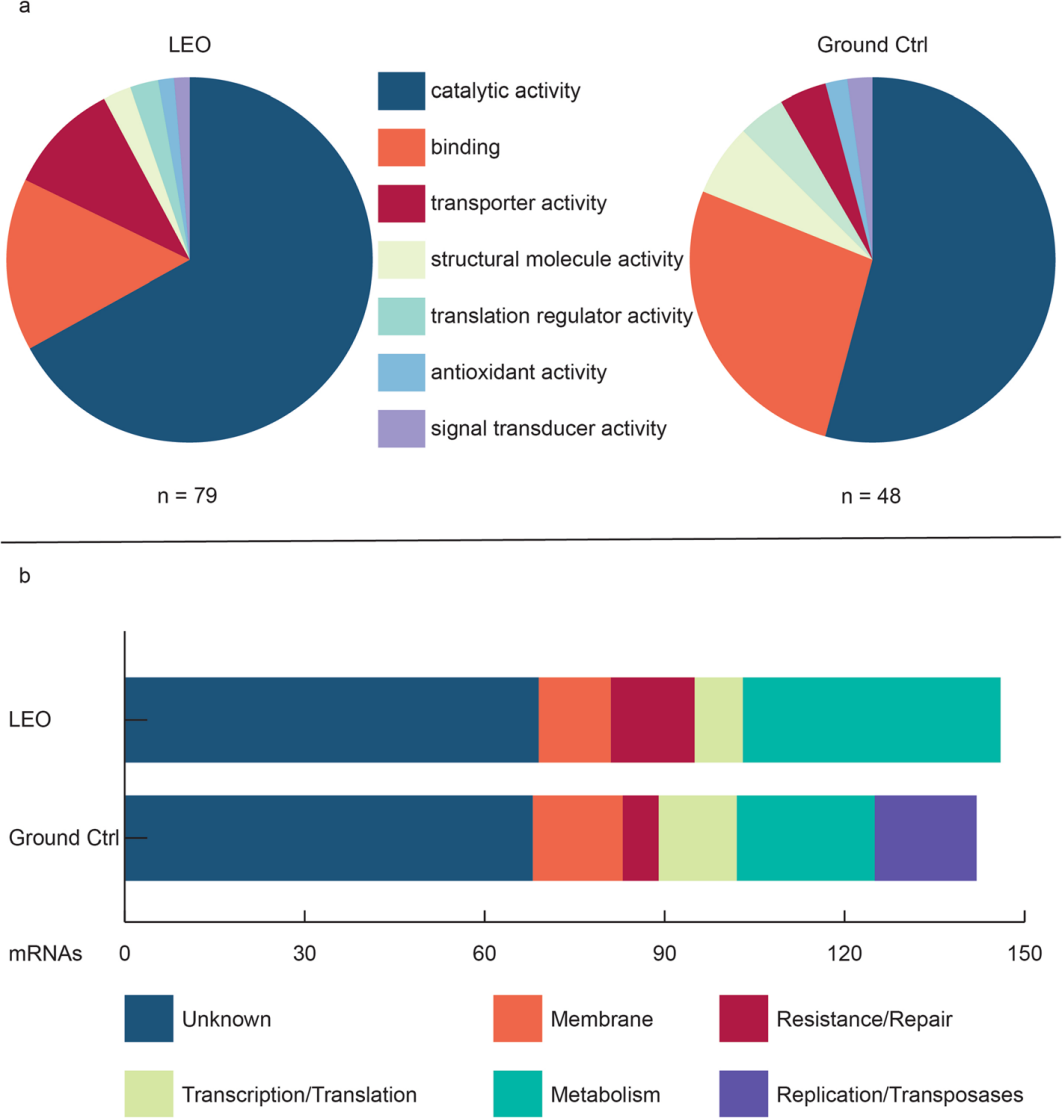
Annotation of statistically relevant proteomic and transcriptomic data. **a** Molecular Function Gene Ontology (GO) annotation of proteins significantly higher or lower abundant (*p* value < 0.05) between LEO exposed and ground control (Ctrl) cells. **b** Manual annotation of mRNAs significantly higher or lower abundant (*q* value < 0.05) between LEO exposed and ground control (Ctrl) cells.

**Fig. 6:**
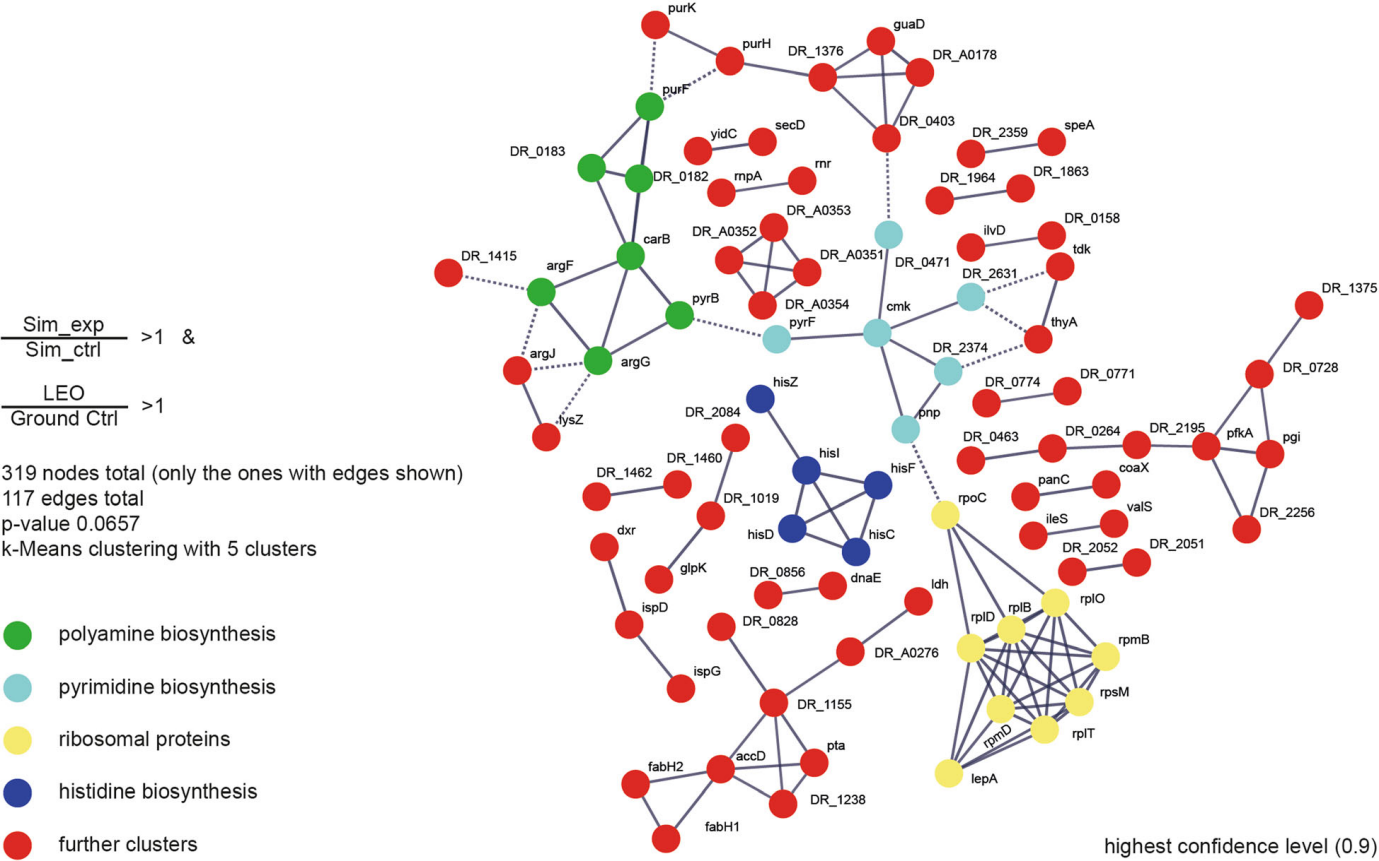
Comparative protein-protein network analysis. Proteins of *D. radiodurans* which were more abundant after LEO exposure compared to ground control and proteins which were more abundant after exposure to simulated LEO conditions compared to corresponding non-exposed control chosen for the network. This list was uploaded to the STRING database. Network construction was performed at highest confidence level (0.9) with k-Means clustering containing five clusters. Annotation was performed according to proteins present in the clusters

**Fig. 7:**
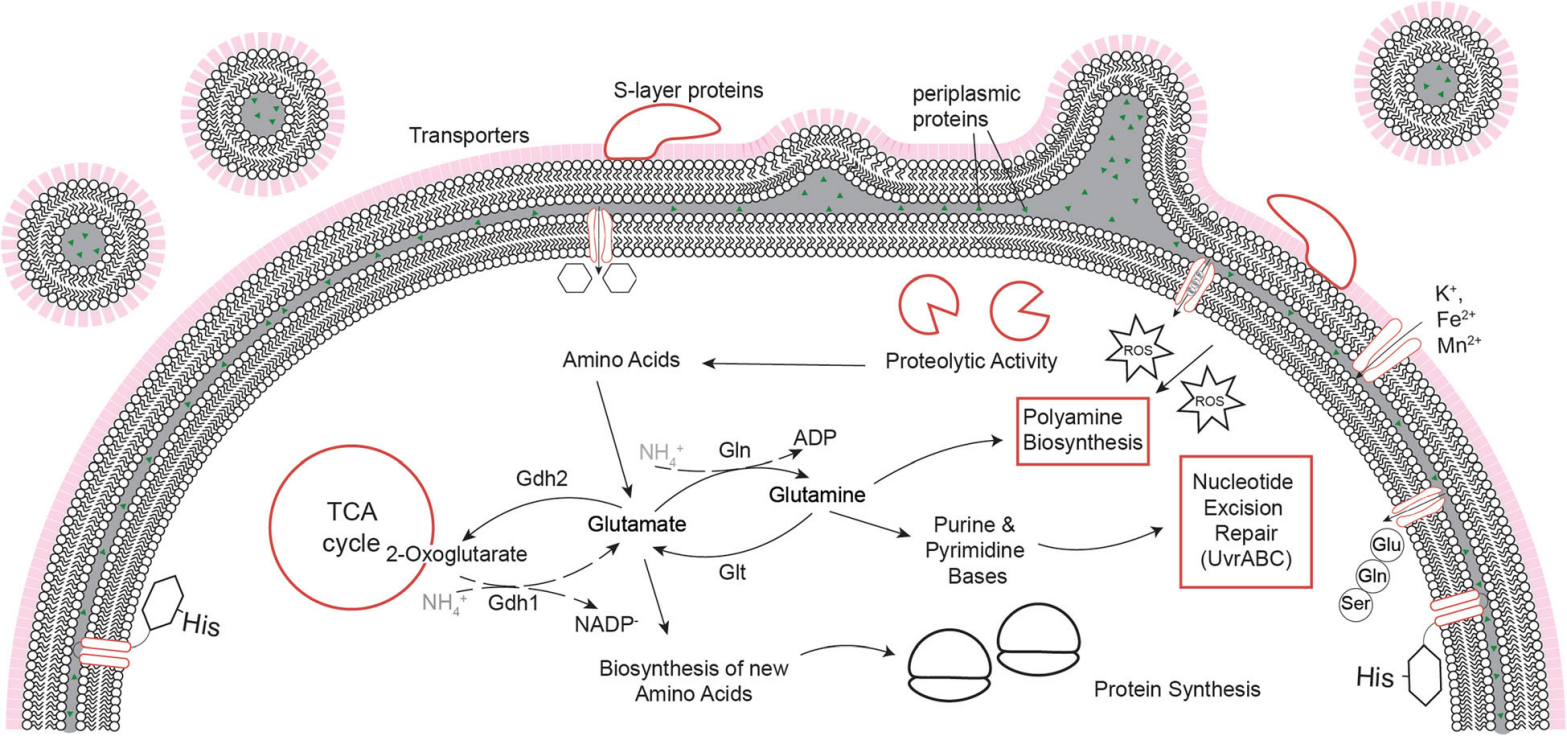
Multi-faceted response of *D. radiodurans* after LEO exposure. Molecular response of *D. radiodurans* after exposure to LEO conditions. Recovered after LEO, *D. radiodurans* cells accumulate transporters, show increased abundance of proteases, nucleotide excision repair proteins, and increased polyamine biosynthesis

Data processing with Maxquant identified 325 proteins in the extracellular compartment of each replicate (Table S1). According to *p* values (below 0.05), 8 proteins were more abundant in the extracellular milieu of space exposed cells and 3 proteins were less abundant (Fig. S4A). Out of the eight proteins which were present in all replicates and identified as more abundant in the space exposed cells, one S-layer array related protein DR_1185 (*p* value 0.0487) and one hemin transporter DR_B0014 (*p* value 0.0332) were identified. The Maxquant data processing resulted in 1828 protein hits for the intracellular compartment (Table S2) throughout all replicates (59 % of the whole *D. radiodurans* proteome). First, a quantitative comparison between controls and space-exposed cells was performed. For the statistical approach, only proteins which were identified in all replicates of both conditions were compared, resulting in 1170 out of 1828. The intensities for these 1170 remaining proteins were scaled and used as loadings for a principle component analysis. Figure S4C shows a clear separation for the two conditions on PC1 level, which explained 40.82 % of the variance in the dataset. The five most influential loadings for PC levels are indicated as black arrows. Although no multiple corrections were performed for the statistical evaluation of the proteomics dataset, the categorical overlap of more abundant proteins (Figs. 3, 4, 5, 6, and 7) indicate a directed proteomic response after LEO exposure.

Welch’s *t* tests were performed to identify proteins with a higher abundance in the control and the space-exposed cells. These resulted in 226 proteins (19.3 %) that were identified in all replicates and differentially expressed (*p* value below 0.05) between the conditions, with 153 being more abundant after LEO exposure and 73 more abundant in the control cells (Fig. S4B). To cluster these proteins in molecular function categories, the online tool of the Gene Ontology Consortium, Panther (V 13.1), was used (Fig. 5a). Seventy-nine hits were found for proteins, which were more abundant after LEO exposure, whereas 48 hits were found for the proteins which were less abundant. The relative distribution between the categories differed between the two groups. Proteins with catalytic activity and transporter activity were more abundant after exposure, while proteins with binding abilities became less abundant (Fig. 5a). Taking a closer look into the categories revealed for transporters and binding, which were more abundant after exposure, proteins such as the two histidine kinases DR_B0029 (*p* value 0.046) and DR_1174 (*p* value 0.047) and the ABC transporter DR_0096 (*p* value 0.031). On the other hand, less abundant proteins do not include any histidine kinases. Furthermore, data obtained after exposure indicated higher abundances of proteins, such as histidine kinases and ABC transporters and S-layer associated proteins, involved in transporter and regulatory activities (Fig. 4a). As shown in Fig. 3d, many proteins involved in the biosynthetic pathway from glutamine to putrescine appear more abundant after exposure to LEO conditions. Finally, most proteins responsible for the ABC endonuclease excision repair mechanism appear in higher abundances (Fig. 4b).

In an attempt to screen for overlaps between real LEO exposure and simulated exposure, proteomics results after both experiments were compared and overlaps were mapped via the STRING (V 11.0) database and illustrated in Fig. 6. Despite proteins responsible for histidine and pyrimidine biosynthesis, the resulting cluster shows that both approaches are indicating an increase of abundance in proteins involved in polyamine biosynthesis.

### *D. radiodurans'* transcriptional response

Transcriptome changes were subsequently compared in response to LEO exposure. Out of 3099 identified mRNAs (Table S3), 146 were more abundant in cells which were exposed to LEO and 142 were less abundant after LEO exposure (Fig. S4D). log_2_ fold changes $\frac{\mathrm{LEO}}{\mathrm{Ctrl}}$ ranged from − 4< *x* < 2. For further interpretations, all mRNAs with a *p* value below 0.05 were manually categorized (Fig. 5b). The number of mRNAs which code for proteins connected to resistance/repair and metabolic processes was considerably higher in LEO exposed cells. However, mRNAs which code for proteins that are associated with replication were only identified as more abundant in ground control cells. Most of these proteins were transposases, which are associated with gene reshuffling.

For a more detailed analysis, significantly more abundant mRNAs were uploaded to Panther (V 13.1) to cluster for molecular functions, biological processes, cellular components, and corresponding protein classes, respectively (Fig. S5). The analysis revealed mRNAs coding for proteins involved in regulatory and transporter activities. A part of differentially expressed mRNAs in the biological process category are responsible for answering stimuli, for instance caused by environmental stresses, involving the two catalases *katA* (*q* value < 0.002) and *DR_A0259* (*q* value < 0.002). Regarding energy metabolism, the protein class pie chart includes pyruvate dehydrogenase *DR_0257* (*q* value < 0.002) in the category of metabolite interconversion enzymes. The cellular component category membrane contains a ferrous iron transporter *DR_1219* (*q* value < 0.002). Further mRNAs, representing the transporter category showed certain overlaps with the proteomics approach, such as histidine kinases and several ABC transporters (Fig. 4a).

Although not to the same extend as shown in the proteomics analysis, some mRNAs for the biosynthesis of the polyamine putrescine from glutamine appeared more abundant after LEO exposure (Fig. 3d).

### *D. radiodurans'* metabolomic response

Primary metabolites were measured after recovery of LEO exposed cells and control cells. Results of the targeted analysis are presented in Fig. 3a, b and Table S4. In general, most metabolites were more abundant in controls compared to cells after LEO exposure (Fig. 3). TCA cycle-related metabolites, e.g., pyruvic acid, succinic acid, and fumaric acid were also more abundant in control cells. Amino acids showed a similar pattern throughout the samples. Stress molecule polyamine putrescine is the only metabolite higher represented after LEO exposure compared to the ground control (Fig. 3b). In case of the untargeted GC metabolomics approach, 68 peaks were annotated by one of the three databases used (Table S5). Untargeted analysis revealed that sugars identified via databases were less presented in LEO exposed cells upon recovery (Fig. 3c).

Finally, a comparative analysis of the primary metabolism between the simulation experiment and the LEO exposed cells is illustrated in Fig. S6. In general, both exposures led to similar results: amino acids seem to be less abundant after exposure, although this trend is particularly more visible after the simulation experiment.

## Discussion

The current findings based on integrated −omics techniques supported by EM-assisted analysis provided a better understanding of molecular mechanisms of the complex rewiring which cells experience during early stages of recovery from the LEO environment. Consistent with the extreme radiation and dehydration resistance of *D*. *radiodurans*, no detectable damage of the cell surface and morphology was observed after LEO exposure (Fig. 1). On a molecular scale, effects of the LEO environment were mirrored in several layers. Results of transcriptomic, proteomic, and metabolomic measurements of space exposed cells indicated a cellular focus on repair mechanisms and metabolization of exogenous resources at the early stage of recovery. Although responses on different −omics levels intertwine, stress-induced changes in the transcriptome and metabolome might appear quicker compared to the proteomic level. Due to the limited, precious space returned material, provided results give a snapshot impression after 2 h of recovery in complex medium.

Results presented in this study may increase awareness regarding planetary protection concerns on, for instance, the Martian atmosphere which absorbs UV radiation below 190–200 nm [35]. To mimic this condition, our experimental setup on the ISS included a SiO_2_ glass window, which blocks out UV light < 190 nm. The term planetary protection refers to protecting celestial bodies from contamination by terrestrial life and protecting Earth from contamination from other celestial bodies which may return with samples taken from other Solar System bodies [36]. In particular, spore-forming bacteria, such as *Bacillus subtilis* 168 and *Bacillus pumilus* SAFR-032 gained attention during the EXPOSE-E mission on the ISS [37]. As approximately 50 % of spores survived the exposure, generated data demonstrated a high chance of survival of spores on a future Mars mission. As our experiment results showed that the non-sporulating bacterium *D. radiodurans* is similarly capable of surviving at LEO conditions for 1 year (Fig. 1), it is necessary to consider the obtained data for future sterilization operations before space missions. Combining survival studies with electron microscopy (Fig. 2) and different −omics studies (Figs. 3, 4, 5, and 6) allowed us to understand how *D. radiodurans* can survive such harsh conditions over a long-term period.

### Proteometabolic rearrangements as response to the low Earth orbit environment

The overall amount of free amino acids, organic acids (TCA cycle intermediates), and sugars decreased in LEO exposed cells. Although limited under space-simulated conditions, previous studies have shown a similar tendency after exposure of *D*. *radiodurans* to UVC radiation combined with vacuum and solely vacuum [23, 24]. Our untargeted metabolomics approach indicated a reduced level of sugars during repair after LEO exposure (Fig. 3c). Sugars can be used as a carbon and energy source, which in turn can be utilized by various repair mechanisms, for instance the repair of damaged nucleic acids.

*D. radiodurans* are organotrophic bacteria which possess proteolytic properties for protein degradation and amino acid catabolism. In 2010, Daly et al. [29] demonstrated an induction of their proteolytic activity following ionizing radiation. Our proteomics dataset after LEO exposure supports this assumption. Based on proteins identified in all replicates, nine proteases were found at higher abundances after LEO exposure, two of them significantly increased (Fig. S4E). Amino acids are used as nitrogen and carbon source, which can be further utilized for many metabolic processes, e.g., growth and nucleic acid repair. As *D. radiodurans* is not able to utilize ammonia as a nitrogen source [38], it completely relies on exogenous amino acids, and this elevated pull of proteases presumably aims to degrade damaged proteins in order to deliver more amino acids during recovery from LEO exposure. The relative amount of amino acids (Fig. 3a) differs most between ground control and space exposed cells for glutamine and glutamic acid, as more of these amino acids are needed for nucleic acid repair mechanisms after space exposure [38]. Glutamine and glutamate certainly play an important role in growth, but in case of alleviation from stress, which cells received from LEO exposure, results indicate that they might be utilized as intermediates for repair processes. Similar results were observed after exposure to simulated LEO conditions (Fig. S6). Simulated LEO conditions likewise reduced the abundance of most amino acids and organic acids throughout the early repair stage, with glutamine and glutamate showing a strong response (Table S6).

### How to cope with space induced DNA damage?

*D. radiodurans* does not possess any special mechanisms that prevents it from nucleic acid damage, such as double-strand breaks after ionizing gamma irradiation and the amount of radiation-induced double-strand breaks is fairly similar between *E. coli* and *D. radiodurans* [28]. Preliminary studies [21, 39] showed that dehydrated *D. radiodurans* cells are damaged by vacuum and temperature cycles in the LEO. However, UV radiation (100 to 280 nm) is most problematic for survival of *D. radiodurans* cells. On Earth, harmful solar UVC radiation is not dangerous to biota, because of an oxygen and ozone shield in the Earth’s atmosphere. LEO, however, does not provide such defense possibilities. Therefore, dried cells outside the ISS were protected from most deleterious UVC radiation below 200 nm by a SiO_2_ glass window. Apart from breaks in the DNA, UV radiation can cause bipyrimidine photoproducts, which later lead to mutations as they interfere with DNA replication [40]. Usually, UV induced lesions can be repaired by photoreactivation, which uses energy from visible light to enzymatically remove pyrimidine dimers [41]. However, as the necessary enzyme is not present in *D. radiodurans* [42], damages, caused by solar UV radiation on dehydrated *D. radiodurans* cells are repaired by nucleotide excision repair. Truglio et al. [32] already recognized the flexibility of the UvrABC excision repair mechanisms in prokaryotes, which is very well suited to repair damage caused by UV radiation. Our proteomics data (Table S2) shows that all key proteins involved in the UvrABC mechanism, which are responsible for detecting lesions and cutting DNA are more abundant after exposure (Fig. 4b). Apparently, there are two UvrA proteins in *D. radiodurans*, which transport UvrB to the damaged DNA. Usually, the intracellular concentration of UvrB is much higher than UvrA, as one UvrA dimer can transport multiple UvrB proteins onto different damage sites [32]. The second UvrA protein might accelerate transport as it increases the specificity to identify target regions. Apart from both UvrA proteins (both *p* value 0.0065), also UvrB (*p* value 0.0100) and UvrC (*p* value 0.0141), which are responsible to cut out a 12 or 13-mer with the DNA lesion, were more abundant after space exposure (Fig. 4b). Finally, the transcription repair coupling factor Mfd (*p* value 0.0075) showed an increase in abundance after LEO exposure. In bacteria, Mfd scans DNA to find RNA polymerases which are blocked by strand lesions. Upon detection, it mediates the release of the stalled RNA polymerase and recruits the nucleotide excision repair machinery to the damaged site [43]. Furthermore, both simulated LEO exposure and real LEO exposure showed an increased abundance of proteins involved in pyrimidine biosynthesis (Fig. 6; S2 and Table S7). Many organisms require efficient pyrimidine biosynthesis for rapid cell proliferation and adaption to cell stress [44–46]. We propose a role of pyrimidine biosynthesis in general stress response of *D. radiodurans*; however, unravelling the exact mechanisms require further experiments on mutants under pyrimidine depleting conditions during stress exposure.

### The molecular stress reactions after low Earth orbit exposure

Exposure to extreme environmental conditions, as those present in LEO, is very deleterious to nucleic acids. If the UvrABC mechanism proteins are still intact, space induced DNA damage can be repaired while the recovered cells seem to be still in a metabolically inactive state. Transcriptomic and proteomic measurements revealed an increase of transporter proteins after space exposure (Fig. 4a). These transporters might be used for external nutrients (e.g., amino acids, sugars, and metal cofactors), which are necessary for repair processes. At the same time, EM-based observations of *D*. *radiodurans* recovering after LEO exposure revealed intensive vesicle trafficking associated with the outer membranes (Fig. 2). Based on our proteo-transcriptomic approach, such intensified membrane-associated trafficking may reflect higher levels of membrane-bound molecular machinery of LEO exposed *D*. *radiodurans* (Fig. 4a). Intensified vesiculation after recovery from LEO exposure can serve as a quick stress response, which augments cell survival by withdrawing stress products (e.g., damaged or misfolded proteins) [47]. Additionally, outer membrane vesicles may contain proteins important for nutrient acquisition, DNA transfer, transport of toxins and quorum sensing molecules [48, 49], eliciting the activation of resistance mechanisms after space exposure. In this regard, the exact role of intensified vesiculation in LEO exposed *D*. *radiodurans* is a topic that deserves more attention and thorough analysis in the future.

The ground control cells, which did not suffer exposure damage, showed an increase of transcripts, which code for replication proteins and transposases (Fig. 5b). Control cells are already closer to the exponential phase than the space exposed cells. Proteomics data supports this assumption, as ribosomal proteins and proteins involved in folding of new proteins were more abundant in the control cells, to enable upcoming cell replication. Three ribosomal proteins (RlmN, RpmC and RtcB) were exclusively found in all three control replicates (Table S2).

One cause of the extreme resistance of *D*. *radiodurans* against radiation and oxidative damage is based on the high levels of constitutively expressed catalase and superoxide dismutase activity [50]. These enzymatic systems are devoted to the protection of cells against toxic reactive oxygen species. Our transcriptomic analysis revealed that genes coding oxidative resistance proteins, such as the catalases *katA* (*q* value < 0.002) and catalase *DR_A0259* (*q* value < 0.002), are more abundant in LEO exposed cells (Table S2). Previous proteomics studies showed an overrepresentation of proteins involved in the oxidative defense system after ionizing radiation was applied [51, 52]. Obviously, upon recovery from LEO exposure, *D. radiodurans* realized a potential oxidative threat and prioritized transcribing oxidation response proteins. The gene *PdxT* and its protein product “redox active pyridoxal 5′-phosphate synthase” are both overrepresented after LEO exposure in our proteo-transcriptomic analysis (Fig. 3d). We previously reported the elevated expression of the pyridoxine biosynthesis proteins *PdxT* and *PdxS* in *D*. *radiodurans* in response to space-related stress stimulus (UVC and vacuum) [23]. The enzymes of pyridoxal 5′-phosphate biosynthesis are singlet oxygen resistance proteins involved in the synthesis of vitamin B_6_, an efficient singlet oxygen quencher and a potential antioxidant [53].

Furthermore, metabolomic analysis of LEO exposed cells revealed a higher abundance of polyamines (e.g., putrescine), which potentially function as primordial forms of stress molecules [54] (Fig. 3b). Additionally, according to proteomic and transcriptomic analyses, several genes/proteins connected to putrescine biosynthesis were more abundant after LEO exposure (Fig. 3d). Furthermore, several proteins involved in this pathway were also identified as more abundant after simulated LEO conditions (Fig. 6). This indicates that polyamines are used as general stress response molecules during recovery of *D. radiodurans* from space exposure.

## Conclusion

In summary, our data provides molecular evidence for a multi-faceted response of *D. radiodurans* after LEO exposure (Fig. 7) by combining proteomic, transcriptomic, and metabolomic analyses with electron microscopy tools. We conclude that the increased abundance of proteins related to the cell envelope associated transport machinery along with intensive vesiculation may alleviate the stress response of *D. radiodurans* during early recovery after LEO exposure. These molecular strategies can facilitate not only nutrient uptake, but also cellular waste removal, distribution of solute, and trafficking of potential signaling molecules. Additionally, our observations point to *D. radiodurans’* utilization of putrescine and ROS scavenging proteins (e.g., catalases) as important determinants in antioxidant defense mechanisms to cope with outer space-induced oxidative damage. Ultimately, the UvrABC endonuclease excision repair mechanism is likely to be recruited to repair nucleic acid damage. Conclusively, the results suggest that survival of *D. radiodurans* in LEO for a longer period is possible due to its efficient molecular response system. This indicates that even longer, farther journeys are possible for organisms with such capabilities.

## Experimental design

### Tanpopo mission exposure

For this space exposure experiment, *Deinococcus radiodurans* (ATCC 13939) cells were placed inside wells of aluminium plates until 1000 μm of cell layers were reached, desiccated, and positioned inside an exposure panel designed by the Tanpopo space mission team [33]. To reach the required thickness of cell layers, multiple 3 μL aliquots of cell suspension containing ~ 10^8^ cells/mL were added and air-dried. The cell suspension was taken from an overnight culture, grown in TGB medium (1 % tryptone, 0.2 % glucose, 0.6 % beef extract) at 30 °C in an incubator shaking at 150 rpm. SiO_2_ filters were put on top of the plates to cut off harmful UV radiation shorter than 200 nm [55]. The exposure panels were on board the SpaceX Dragon commercial cargo spaceship, which launched on 15 April 2015 from Cape Canaveral (USA) by the Space-X Falcon-9 rocket. They were manually attached to the exposed experiment handrail attachment mechanism (ExHAM) on the Japanese exposure facility of the International Space Station, which was transferred to its final position on 26 May 2015. For 1 year, samples were exposed to a total UV fluence of 3.1 × 10^3^ kJ/m^2^ (200–315 nm), total cosmic radiation of 250–298 mGy, temperature fluctuations between – 21.0 ± 5 °C and 23.9 ± 5 °C, pressure between 10^–7^ Pa and 10^–4^ Pa and 0 % humidity. Space-exposed (LEO) cells returned after 1 year on 26 August 2016 on board the SpaceX Dragon C11, which landed in the Pacific Ocean. Ground control cells (Ground Control) were prepared simultaneously and stored in a desiccator during all exposure time.

### Exposure to simulated LEO environmental factors

*D. radiodurans* cells deposited in dried form on Tanpopo exposure plates as for the mission were exposed in PSI 5 of the Astrobiology Space Simulation facilities at DLR Cologne equipped with a SiO_2_ window and a temperature control plate to a final pressure of 10^−5^ Pa for 90 days (Sim_exp). Samples were in parallel exposed to UV radiation of 3.4 × 10^3^ kJm^−2^ with wavelengths > 200 nm from a solar simulator SOL2 (Dr. Hönle GmbH) and temperature cycles from – 21 °C to + 24 °C by an attached cryostat (Lauda). Unexposed simulation controls (Sim_ctrl) were stored inside a desiccator with silica gel at 21 °C.

### Survival assays

To evaluate survivability of the LEO returned cells, colony forming units were counted on plates and compared to the growth of ground control cells. After exposure, dehydrated cells were recovered from the aluminium plate wells by resuspending the cell pellet in sterile phosphate buffer (137 mM NaCl, 3 mM KCl, 10 mM Na_2_HPO_4_, 2 mM KH_2_PO_4_). Suspensions were serially diluted in phosphate buffer and dropped onto TGB plates. Colonies were counted after incubation at 30 °C for 36 h. Surviving cell fractions were determined as *N*/*N*_0_, where *N* was the number of colony-forming units remaining after space exposure or 1 year in the desiccator (ground control) and *N*_0_ was that at the time of cell preparation 1 year before.

### Recovery conditions and preparations for multi-omics analyses

In total, three biological replicates of LEO exposed cells and ground control cells were recovered and used for –omics analysis. Each replicate contained the amount of cells from one aluminium well. In case of the simulation experiment, four replicates from the exposed (Sim_exp) and control cells (Sim_ctrl) were used for analysis. For each biological replicate, the content of two wells with *D. radiodurans* cells (each 1000 μm thickness of cell layer) was resuspended in 30 mL of TGB medium. OD_600_ was measured for each replicate before incubating the cells at 30 °C, 150 rpm for 2 h. After 2 h of incubation, OD_600_ was measured again (Fig. 1) and cells were harvested by centrifugation at 3000×*g*/2 min/4 °C. The supernatant was kept for a subsequent analysis of proteins, present in the extracellular compartment. For washing, the pellets were resuspended in 20 mL sterile, ice cold PBS, and centrifuged at 3000×*g*/2 min/4 °C. The supernatant was discarded, pellets were resuspended in 1.5 mL ice cold PBS, and centrifuged at 1500×*g*/2 min/4 °C. For the final washing step, 1.5 mL PBS was added and the cell suspension of each well was separated in a 500 μL aliquot for metabolite extraction and a 1000 μL aliquot for protein and RNA extraction. After centrifugation at 1500×*g*/2 min/4 °C, the supernatant was discarded, cells were snap frozen in liquid nitrogen and stored at − 80 °C overnight until further extraction. The protocol used for the simultaneous extraction of mRNA, proteins and metabolites was previously established by Weckwerth et al. (2004) [56]. The workflow was optimized for the space experimental setup in simulation experiments prior to the LEO exposed samples [23, 24, 57].

### Integrative extraction and measurements of biological molecules

#### Polar metabolite extraction

Cells from each well were resuspended in 1 mL methanol/chloroform/water (2.5:1:0.5), and ceramic beads were added to the samples. Homogenization was performed with a MagNA-Lyser (Roche) 5 times at 7000 rpm/30 s with cooling in between the cycles. Samples were incubated 15 min on ice, centrifuged at 21000×*g*/6 min/RT, and the supernatant was transferred into new tubes. Two hundred microliters of water was added to the supernatant to achieve a phase separation. Samples were centrifuged at 10000×*g*/5 min/RT and the upper, polar phase was transferred into a new tube. The polar phase was put under constant nitrogen steam at 35 °C until total dryness. Samples were frozen at − 20 °C until further analysis.

#### Analysis of polar metabolites with GC-TOF

Before injection to the GC-TOF, samples were derivatized. Derivatization, further sample preparation steps and instruments for measurements were described previously [24].

Identifications of metabolites were based on comparing the obtained spectra to those of a standard mix, containing several important primary metabolites with ChromaTOF. For quantification, a high abundant, preferably unique mass for each metabolite was selected and the peak on the chromatogram was integrated. Areas were normalized to OD_600_ values which were measured before the extraction.

#### RNA extraction

For extraction of RNA, cells were resuspended in 1000 μl QIAzol together with ceramic beads (Roche, Basel, Switzerland; MagNA Lyser Green Beads, CatNo 03358941001). Homogenization was performed as described above. After cell disruption, samples were incubated for 5 min at RT on a rotating wheel and afterwards 200 μL chloroform were added to each sample. After 3 min incubation at RT on the rotating wheel, samples were centrifuged at 21000 *g*/15 min/4 °C to separate phases. The upper, polar phase with the RNA was transferred into a new tube for purification with the RNeasy Lipid Tissue kit according to the manufacturer’s manual including DNAse treatment. Samples were eluted with 20 μL TE buffer (pH 8) and stored at − 80 °C until further analysis.

#### Analysis of mRNA with Illumina HiSeq

The purified, total RNA was quantified on a NanoDrop (Fig S4) and subsequently measured on a Bioanalyzer BA2100 (Agilent; Foster City, CA, USA) to calculate RNA integrity numbers (RIN numbers of all replicates around 3). rRNA was depleted with the MICROBExpress™ Bacterial mRNA Enrichment Kit using 100 ng total RNA as input, followed by library construction with the NEB® Ultra™ RNA Library Prep Kit for Illumina according to the manufacturers’ manuals. The samples were measured on an Illumina HiSeq instrument at the Vienna BioCenter Core Facilities (VBCF). Results, provided by VBCF as .bam-files, were converted to fastq with bedtools2 [58] and mapped via bowtie2 [59] on the updated genome of *D. radiodurans* type strain R1 [60]. Annotation according to the reference genome and differential gene expression calculations were done with CUFFDIFF [61].

The transcriptomics data for this study have been deposited in the European Nucleotide Archive (ENA) at EMBL-EBI under accession number PRJEB40352.

#### Intracellular protein extraction

After QIAzol extraction and phase separation, the lower, phenolic phase was transferred into a fresh tube for protein purification. To further wash the samples, 550 μL of H_2_O were added to the samples, centrifuged at 10000 *g*/5 min/RT and the lower phase was transferred into a new tube. To precipitate the proteins, 1.5 mL of 0.1 M NH_4_Ac in MeOH (with 0.5 % 2-mercaptoethanol) were added and the samples were put at − 20 °C overnight. On the following day, proteins were centrifuged at 10000×*g*/15 min/4 °C. The pellets were subsequently washed three times with acetone and centrifuged at 10000×*g*/5 min/4 °C. After the final washing step, pellets were air dried and stored at − 20 °C until further analysis.

#### Extracellular protein extraction

To identify proteins represented in the extracellular milieu, the extracellular medium/supernatant of each replicate was analyzed. To remove remaining bacteria first, the supernatant samples were filtered through a 0.22 μm membrane (Watson). To each sample, trichloroacetic acid was added to a final concentration of 10 % (V/V) and samples were incubated on ice for 2 h. To pellet the proteins, samples were centrifuged at 38000×*g*/30 min/4 °C and the supernatant was discarded. Pellets were washed three times with acetone and centrifuged at 10000×*g*/5 min/4 °C. After the final washing step, pellets were air dried and stored at − 20 °C until further analysis.

#### Analysis of proteins with LC-Orbitrap

Further sample preparation steps as digestion, peptide purification, and peptide desalting were performed as described previously [23]. For the intracellular compartment, peptides were normalized with a fluorometric peptide assay (Pierce). LCMS analyses were performed using a single-shot LCMS approach with 120 min gradient using a Dionex Ultimate 3000 system (Thermo Fisher Scientific) coupled to a Q-Exactive Plus mass spectrometer (Thermo Fisher Scientific, Germany) with LCMS parameters as described previously [62].

Data analysis was performed with Maxquant [63] with following settings: min 7 amino acid mass for peptide identification, min 1 unique peptide for protein identification, label-free quantification (LFQ), max missed cleavages 2, max number of modifications per peptide 5, and cuts after proline are allowed.

The mass spectrometry proteomics data have been deposited to the ProteomeXchange Consortium via the PRIDE partner repository with the dataset identifier PXD020047.

#### SEM/TEM analysis

The morphology and cellular integrity of the dehydrated cells of *D. radiodurans* deposited on aluminum plates were examined with a Zeiss Supra 55 VP scanning electron microscope. The dehydrated cells were coated with a thin Au/Pd layer (Laurell WS-650-23 spin coater). The imaging of dehydrated clustered cell layers was performed with an acceleration voltage of 5 kV. For electron microscopy analyses of recovered cells after LEO exposure*, D. radiodurans* cells were sampled at 0 and 120 min after recovery, washed three times in PHEM-buffer (360 mM PIPES, 150 mM HEPES, 60 mM EGTA, 12 mM MgCl_2_) and subsequently fixed in the same buffer containing 2.5% glutaraldehyde. After the second wash cycle post-fixation was carried out using 1% OsO_4_ in H_2_O and pellets were dehydrated in a graded series of ethanol. For transmission electron microscopy (TEM), pellets were transferred to dried acetone for subsequent infiltration and embedding in epoxy resin (Agar Scientific, Low Viscosity Resin Kit). Ultrathin sections (50–70 nm) were mounted on TEM support grids (Agar Scientific, copper, 200 mesh hex) coated with formvar film, stained with gadolinium triacetate [64] and lead citrate [65], and finally examined in a Zeiss Libra 120. For scanning electron microscopy analysis (SEM), samples were treated equally (washed, fixed, EtOH dehydrated) until TEM samples were transferred into dried acetone. Pellets for SEM examination were spread on filters (Whatman, 13 mm, 0.2 μm pore size), dried via critical point drying (Leica EM CPD 300), mounted on stubs and coated with gold (JEOL JFC 2300 HR). Samples were viewed with a JEOL IT 300.

#### Quantification and statistical analysis

Statistical evaluation of transcriptomics data was based on fold changes and *t* tests including multiple testing correction (Benjamini-Hochberg correction) results calculated by CUFFDIFF. Relative comparison of proteomics data was based on the LFQ intensities calculated by Maxquant. To compare exposed and control samples, fold changes were calculated (mean_exp_/mean_ctrl_) and Welch’s *t* tests were performed. To compare metabolomics data, the peak areas calculated by ChromaTOF were normalized to OD_600_ values of the corresponding sample. Afterwards, fold changes and *p* values (Welch’s *t* test) for all identified metabolites were calculated. Due to the limitation in replicates and consequently high standard deviations, no multiple testing corrections were performed for proteomics and metabolomics analyses as this would drastically increase stringency on *p* value requirements. However, providing these unique datasets after applying LEO exposure is obligatory and although the significance of the statistical evaluation might be limited (considering a 95 % significance level), looking at the datasets in combination with fold changes and the corrected *p* values from the transcriptomics approach can lead to valid conclusions.

## Supplementary information

**Additional file 1: Figure S1**. SEM images of LEO exposed and control cells. Scanning electron microscopy (SEM) images showing upper surface of multilayers of dehydrated cells of *D. radiodurans* deposited on aluminum plates. (a, c) SEM images of *D. radiodurans* cells exposed to LEO in Tanpopo mission. (b, d) SEM images of ground control cells of *D. radiodurans*. **Figure S2**. High magnification SEM images of LEO exposed cells. Higher magnification SEM images displaying upper surface of multilayers of dehydrated *D. radiodurans* cells after LEO exposure. **Figure S3**. SEM and TEM images after recovery. Scanning and transmission electron microscopy (SEM and TEM) images of *D. radiodurans* cells recovered after LEO exposure in complex medium. (a) SEM image of recovered *D. radiodurans* cells after LEO exposure. (b) TEM image of recovered *D. radiodurans* cells after LEO exposure. (c) SEM image of ground control *D. radiodurans* cells. (d) TEM image of ground control *D. radiodurans* cells. **Figure S4.** Statistical comparison of proteomics data between LEO exposed and control cells. (a) Protein hits present in all replicates, with a p-value below 0.05 identified in the extracellular compartment of LEO exposed and ground control cells. (b) Protein hits present in all replicates, with a p-value below 0.05 identified in the intracellular compartment. (c) PCA of all measured intracellular proteins. (d) Negative decadic logarithm of corrected p-values (q-values, y-axis) versus log_2_ fold change (x-axis) of all measured mRNAs. Transcripts with a q-value below 0.05 and a fold change >|1.5| are emphasized. (e) Abundance of proteases identified in the intracellular compartment. Significant differences are indicated with an asterisk (*). **Figure S5**. Gene Ontology annotation of higher abundant transcripts. Includes Gene Ontology annotation of molecular functions, biological processes, cellular components and protein classes of higher abundant transcripts with a q-value<0.05. **Figure S6**. Comparison of targeted metabolomics approaches. Both charts show the same metabolites. The upper one shows results after simulated LEO exposure of *D. radiodurans* (Sim_exp) compared with corresponding control (Sim_ctrl). The lower one shows results after the real LEO exposure experiment, comparing LEO exposed and ground control (Ctrl) cells. **Table S1**. LEO experiment - Table of raw extracellular protein LFQ intensities, corresponding statistical analysis, the number of identified unique peptides and the calculated Maxquant score. **Table S2**. LEO experiment - Table of raw intracellular protein LFQ intensities, corresponding statistical analysis, the number of identified unique peptides and the calculated Maxquant score. **Table S3**. LEO experiment - Table of calculated FPKM values and corresponding statistical analysis. **Table S4**. LEO experiment - Normalized values for targeted metabolites and corresponding statistical analysis. **Table S5**. LEO experiment - Normalized values for untargeted metabolites, corresponding statistical analysis and library search. **Table S6**. Simulation experiment - Normalized values for targeted metabolites and corresponding statistical analysis. **Table S7**. Simulation experiment - Table of raw intracellular protein LFQ intensities, corresponding statistical analysis, the number of identified unique peptides and the calculated Maxquant score.

## Data Availability

All data used for the manuscript is available in the supplement figures.
