# Supplementary material for: Molecular repertoire of Deinococcus radiodurans after 1 year of exposure outside the International Space Station within the Tanpopo mission
Source: Microbiome. 2020 Oct 29;8:150. doi: 10.1186/s40168-020-00927-5 (PMC7597052; doi:10.1186/s40168-020-00927-5)
Supplement: Supplementary file 2 — Additional file 1: Figure S1. SEM images of LEO exposed and control cells. Scanning electron microscopy (SEM) images showing upper surface of multilayers of dehydrated cells of D. radiodurans deposited on aluminum plates. (a, c) SEM images of D. radiodurans cells exposed to LEO in Tanpopo mission. (b, d) SEM images of ground control cells of D. radiodurans. Figure S2. High magnification SEM images of LEO exposed cells. Higher magnification SEM images displaying upper surface of multilayers of dehydrated D. radiodurans cells after LEO exposure. Figure S3. SEM and TEM images after recovery. Scanning and transmission electron microscopy (SEM and TEM) images of D. radiodurans cells recovered after LEO exposure in complex medium. (a) SEM image of recovered D. radiodurans cells after LEO exposure. (b) TEM image of recovered D. radiodurans cells after LEO exposure. (c) SEM image of ground control D. radiodurans cells. (d) TEM image of ground control D. radiodurans cells. Figure S4. Statistical comparison of proteomics data between LEO exposed and control cells. (a) Protein hits present in all replicates, with a p-value below 0.05 identified in the extracellular compartment of LEO exposed and ground control cells. (b) Protein hits present in all replicates, with a p-value below 0.05 identified in the intracellular compartment. (c) PCA of all measured intracellular proteins. (d) Negative decadic logarithm of corrected p-values (q-values, y-axis) versus log2 fold change (x-axis) of all measured mRNAs. Transcripts with a q-value below 0.05 and a fold change >|1.5| are emphasized. (e) Abundance of proteases identified in the intracellular compartment. Significant differences are indicated with an asterisk (*). Figure S5. Gene Ontology annotation of higher abundant transcripts. Includes Gene Ontology annotation of molecular functions, biological processes, cellular components and protein classes of higher abundant transcripts with a q-value<0.05. Figure S6. Comparison of targete [file 40168_2020_927_MOESM1_ESM.zip › 40168_2020_927_MOESM1_ESM/Supplementary Information.docx]

**Supplementary Information**

Molecular repertoire of *Deinococcus radiodurans* after 1 year of exposure outside the International Space Station within the Tanpopo mission

Emanuel Ott, Yuko Kawaguchi, Denise Kölbl, Elke Rabbow, Petra Rettberg, Maximilian Mora, Christine Moissl-Eichinger, Wolfram Weckwerth, Akihiko Yamagishi, Tetyana Milojevic


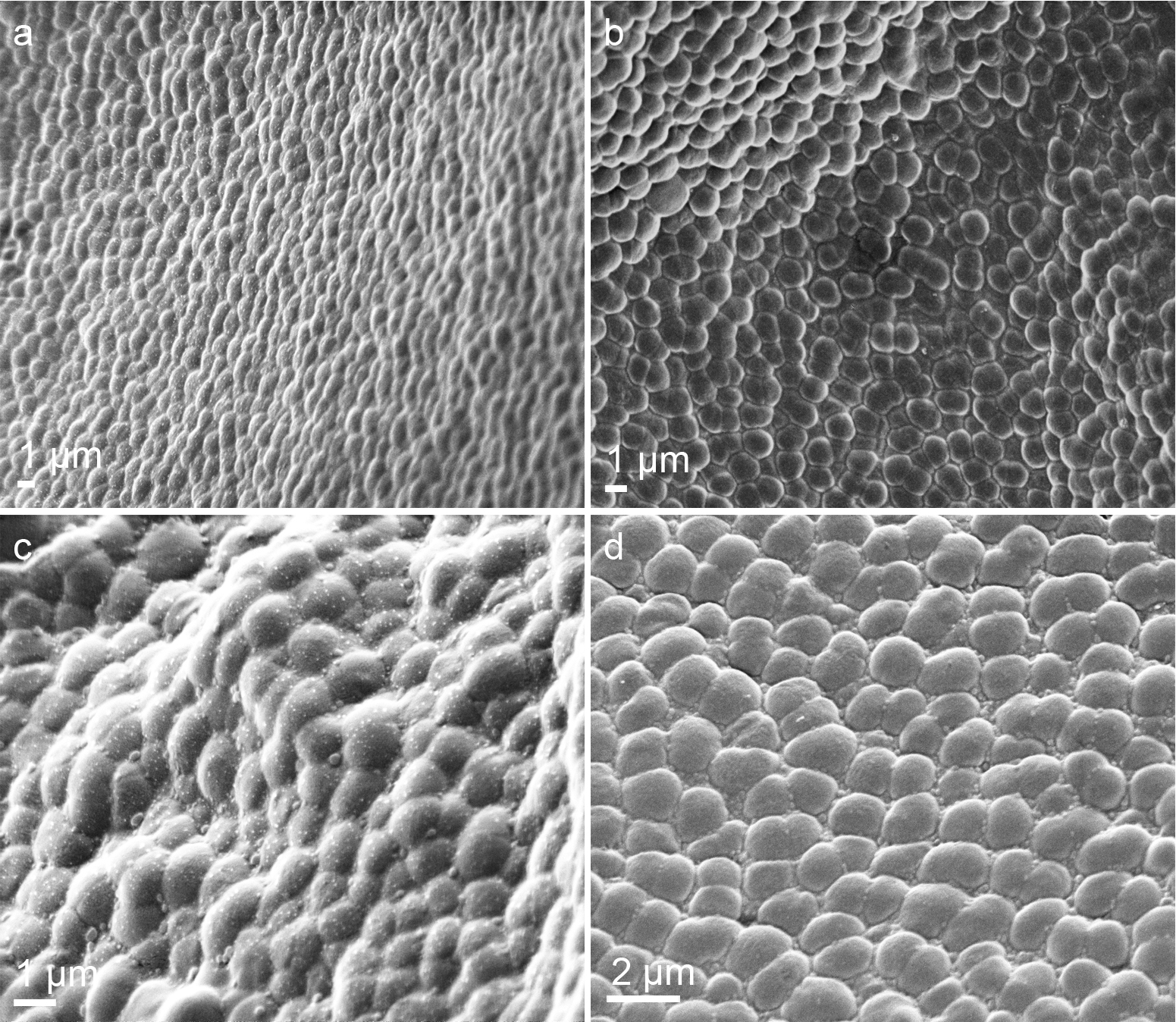


**S1 Fig. SEM images of LEO exposed and control cells.**

Scanning electron microscopy (SEM) images showing upper surface of multilayers of dehydrated cells of *D. radiodurans* deposited on aluminum plates. (a, c) SEM images of *D. radiodurans* cells exposed to LEO in Tanpopo mission. (b, d) SEM images of ground control cells of *D. radiodurans*.

**
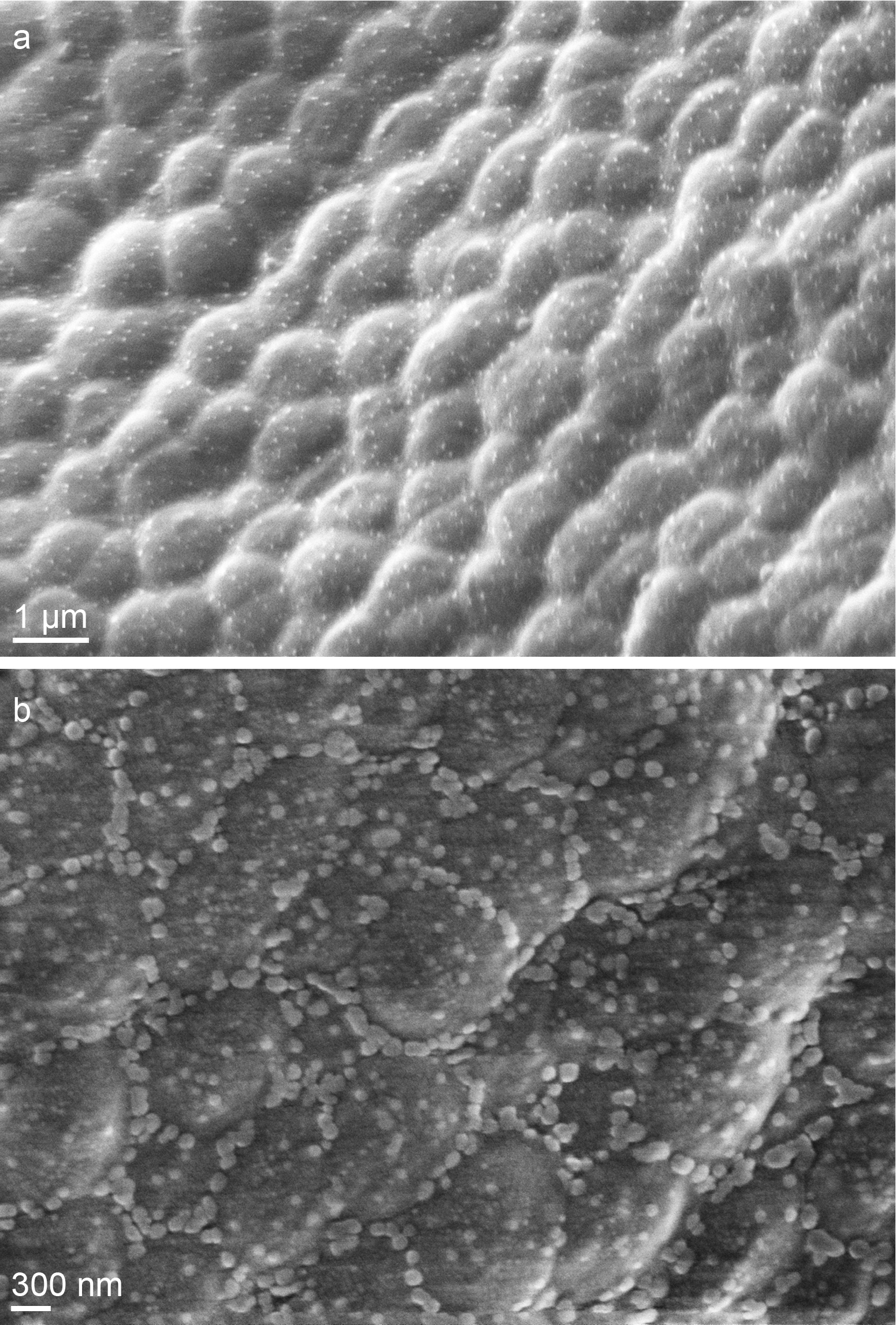
**

**S2 Fig. High magnification SEM images of LEO exposed cells**

Higher magnification SEM images displaying upper surface of multilayers of dehydrated *D. radiodurans* cells after LEO exposure.


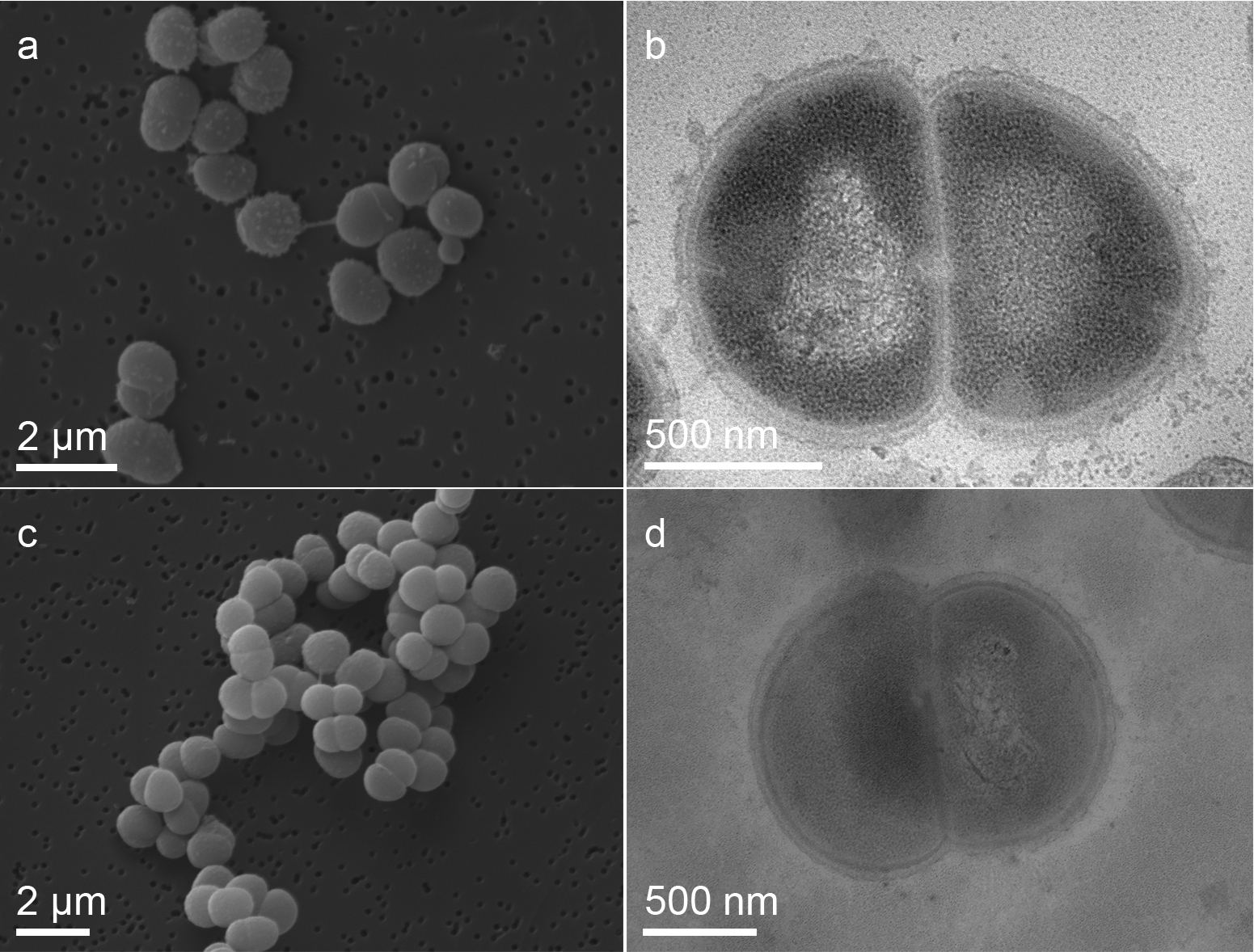


**S3 Fig. SEM and TEM images after recovery.**

Scanning and transmission electron microscopy (SEM and TEM) images of *D. radiodurans* cells recovered after LEO exposure in complex medium. (a) SEM image of recovered *D. radiodurans* cells after LEO exposure. (b) TEM image of recovered *D. radiodurans* cells after LEO exposure. (c) SEM image of ground control *D. radiodurans* cells. (d) TEM image of ground control *D. radiodurans* cells.

**
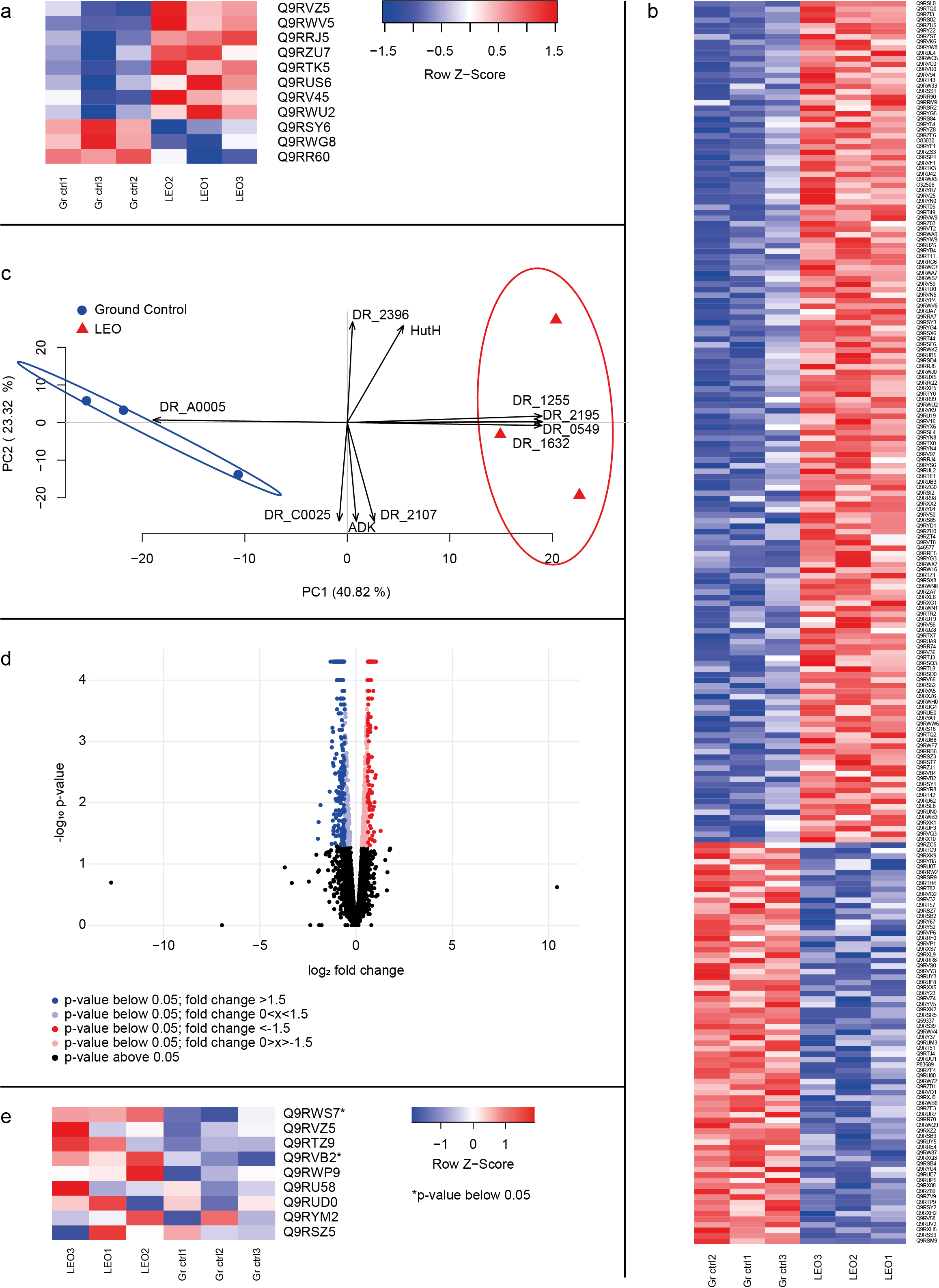
**

**S4 Fig. Statistical comparison of proteomics data between LEO exposed and control cells**

(a) Protein hits present in all replicates, with a p-value below 0.05 identified in the extracellular compartment of LEO exposed and ground control cells. (b) Protein hits present in all replicates, with a p-value below 0.05 identified in the intracellular compartment. (c) PCA of all measured intracellular proteins. (d) Negative decadic logarithm of corrected p-values (q-values, y-axis) versus log_2_ fold change (x-axis) of all measured mRNAs. Transcripts with a q-value below 0.05 and a fold change >|1.5| are emphasized. (e) Abundance of proteases identified in the intracellular compartment. Significant differences are indicated with an asterisk (*).


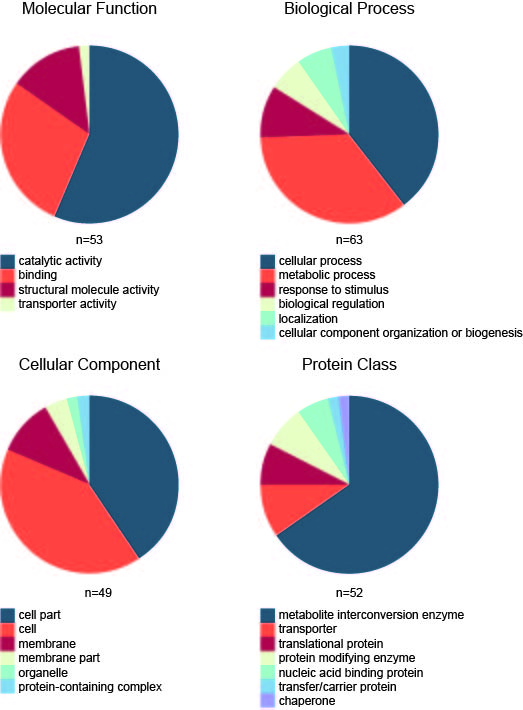
**S5 Fig. Gene Ontology annotation of higher abundant transcripts.**

Includes Gene Ontology annotation of molecular functions, biological processes, cellular components and protein classes of higher abundant transcripts with a q-value<0.05.


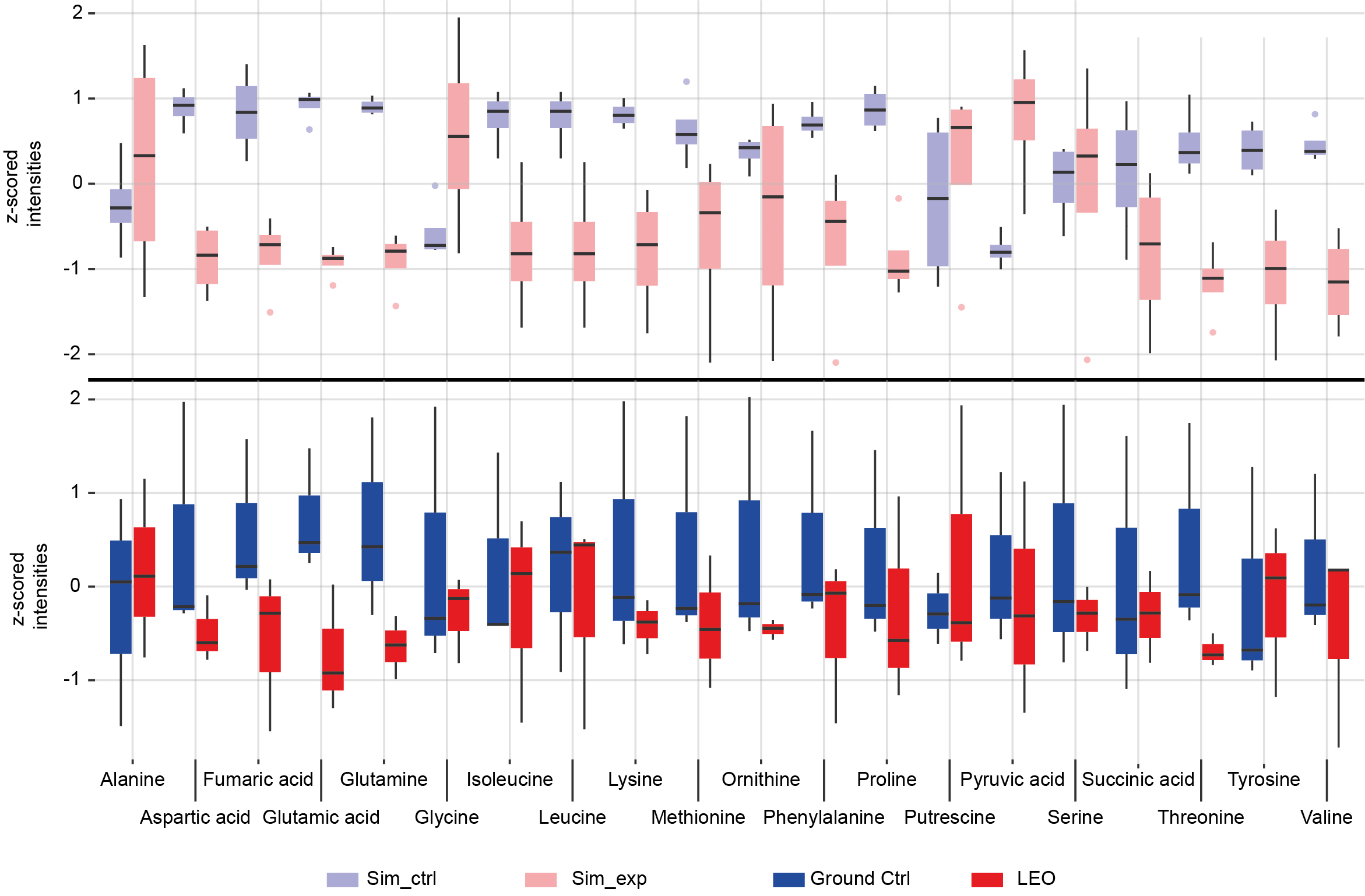


**S6 Fig. Comparison of targeted metabolomics approaches.**

Both charts show the same metabolites. The upper one shows results after simulated LEO exposure of *D. radiodurans* (Sim_exp) compared with corresponding control (Sim_ctrl). The lower one shows results after the real LEO exposure experiment, comparing LEO exposed and ground control (Ctrl) cells.

**S1 Table.**

LEO experiment - Table of raw extracellular protein LFQ intensities, corresponding statistical analysis, the number of identified unique peptides and the calculated Maxquant score.

**S2 Table.**

LEO experiment - Table of raw intracellular protein LFQ intensities, corresponding statistical analysis, the number of identified unique peptides and the calculated Maxquant score.

**S3 Table.**

LEO experiment - Table of calculated FPKM values and corresponding statistical analysis.

**S4 Table.**

LEO experiment - Normalized values for targeted metabolites and corresponding statistical analysis.

**S5 Table.**

LEO experiment - Normalized values for untargeted metabolites, corresponding statistical analysis and library search.

**S6 Table.**

Simulation experiment - Normalized values for targeted metabolites and corresponding statistical analysis.

**S7 Table.**

Simulation experiment - Table of raw intracellular protein LFQ intensities, corresponding statistical analysis, the number of identified unique peptides and the calculated Maxquant score.
